# Identification of Mucormycosis by Fluorescence In Situ Hybridization Targeting Ribosomal RNA in Tissue Samples

**DOI:** 10.3390/jof8030289

**Published:** 2022-03-11

**Authors:** Jill Jasmine Dalimot, Ilka Mc Cormick Smith, Jasmin Gerkrath, Sylvia Hartmann, Oliver A. Cornely, Soo Chan Lee, Joseph Heitman, Volker Rickerts

**Affiliations:** 1 Mycotic and Parasitic Agents and Mycobacteria, Department of Infectious Diseases, Robert Koch Institute (RKI), 13353 Berlin, Germany; jill.dalimot@crick.ac.uk (J.J.D.); mccormick-smithi@rki.de (I.M.C.S.); gerkrathj@rki.de (J.G.); 2 Signalling in Apicomplexan Parasites Laboratory, The Francis Crick Institute, London NW1 1AT, UK; 3 Senckenberg Institute for Pathology, Johann Wolfgang Goethe University Frankfurt, 60323 Frankfurt am Main, Germany; s.hartmann@em.uni-frankfurt.de; 4 Excellence Center for Medical Mycology (ECMM), Department I of Internal Medicine, Faculty of Medicine and University Hospital Cologne, University of Cologne, 50923 Cologne, Germany; oliver.cornely@uk-koeln.de; 5 Cologne Excellence Cluster on Cellular Stress Responses in Aging-Associated Diseases (CECAD), Chair Translational Research, Faculty of Medicine and University Hospital Cologne, University of Cologne, 50923 Cologne, Germany; 6 Clinical Trials Centre Cologne (ZKS Köln), Faculty of Medicine and University Hospital Cologne, University of Cologne, 50923 Cologne, Germany; 7 German Centre for Infection Research (DZIF), Partner Site Bonn-Cologne, 50923 Cologne, Germany; 8 Department of Molecular Genetics and Microbiology, Duke University Medical Center, Durham, NC 27710, USA; soochan.lee@utsa.edu (S.C.L.); heitm001@duke.edu (J.H.); 9 South Texas Center for Emerging Infectious Diseases (STCEID), Department of Molecular Microbiology and Immunology, The University of Texas, San Antonio, TX 78249, USA

**Keywords:** mucormycosis, in situ, hybridization, fluorescence, pathology

## Abstract

Mucormycosis is an invasive fungal infection associated with high mortality, partly due to delayed diagnosis and inadequate empiric therapy. As fungal cultures often fail to grow Mucorales, identification of respective hyphae in tissue is frequently needed for diagnosis but may be challenging. We studied fluorescence in situ hybridization (FISH) targeting specific regions of the fungal ribosomal RNA (rRNA) of Mucorales to improve diagnosis of mucormycosis from tissue samples. We generated a probe combination specifically targeting Mucorales. Probe specificity was verified in silico and using cultivated fungi. Mucorales hyphae in tissue of a mouse model demonstrated a bright cytoplasmatic hybridization signal. In tissue samples of patients with mucormycosis, a positive signal was seen in 7 of 12 (58.3%) samples. However, autofluorescence in 3 of 7 (42.9%) samples impaired the diagnostic yield. Subsequent experiments suggested that availability of nutrients and antifungal therapy may impact on the FISH signal obtained with Mucorales hyphae. Diagnosis of mucormycosis from tissue might be improved by rRNA FISH in a limited number of cases only. FISH signals may reflect different physiological states of fungi in tissue. Further studies are needed to define the value of FISH to diagnose mucormycosis from other clinical samples.

## 1. Introduction

Mucormycosis is an emerging fungal infection diagnosed in diverse patient groups suffering from cancer, diabetes, and trauma and in critically ill subjects, including patients with severe COVID-19 infection. Infections mostly occur after inhalation of fungal spores, leading to localized disease of the respiratory tract presenting as pneumonia or sinusitis. Disseminated infections are mostly diagnosed in immunocompromised hosts, including neutropenic cancer patients, and are associated with high mortality partly because of delayed diagnosis due to unspecific symptoms and lack of sensitive diagnostic tests, and therefore, delayed introduction of specific treatments [1].

Cultivation of Mucorales from clinical samples may not be possible in many cases, and mucormycosis is frequently diagnosed by the presence of typical pauciseptate, broad, ribbon-like hyphae in tissue samples. However, identification of these elements might be difficult and confirmation by molecular tests is recommended for diagnosis of mucormycosis [2].

In situ hybridization targeting ribosomal RNA (rRNA) of microorganisms offers potential as a diagnostic tool for identifying and localizing microbes in an infection process. Due to the quantity of the target structure, rRNA in fungal cells, amplification might not be necessary, leading to specific detection, potentially minimizing the risk for contamination and allowing a localization within a disease process [3]. A study using formalin-fixed, paraffin-embedded tissue (FFPET) samples from patients with mucormycosis demonstrated a high sensitivity and specificity in culture positive samples. However, the method was impaired by high background signal and low intensity or only focal staining in mucormycosis samples [4]. Previous experience with fluorescence in situ hybridization (FISH) targeting rRNA of fungi in FFPET samples from patients with proven fungal infection suggested a potential for rRNA FISH to identify Mucorales in tissue samples, but a specific probe targeting a conserved region of the ribosomal target leading to a good signal intensity when applied to FFPET has not been reported [5].

Typically, short oligonucleotides are used for rRNA FISH for diagnosis of infections. Infections caused by phylogenetically diverse microbes, such as mucormycosis, might be difficult to diagnose by FISH, as single probes may not be sufficient for identifying causative agents due to polymorphisms in the target structure [6].

Here, we explored the use of FISH targeting the rRNA gene of Mucorales for diagnosis of mucormycosis. Probes were designed and evaluated with cultivated fungi targets and non-target fungi. FFPET samples from a mucormycosis mouse model and from patients with proven mucormycosis, as diagnosed by the presence of typical fungal elements in tissue and confirmed by broad-range fungal polymerase chain reaction (PCR) and sequencing if possible, were subsequently used to define the usefulness of FISH for identifying mucormycosis.

## 2. Materials and Methods

### 2.1. Design of FISH Probes Targeting the Ribosomal RNA of Agents of Mucormycosis

FISH probes were constructed as previously described [5]. In short, the 18s rRNA gene was screened for Mucorales-specific sequences in regions known for high signal intensity as evaluated for *Saccharomyces cerevisiae* [7]. Sequences conserved among agents of mucormycosis showing mismatches with other fungi were evaluated using alignments of the fungal 18S rRNA gene in Geneious (Auckland, New Zealand) (Supplementary Tables S2 and S3). A combination of two probes (Mucorales, Lichtheimia) was applied for experimental evaluation by FISH to ensure detection of common agents of mucormycosis. The signal intensity of this probe combination was compared with probe Euk 516, which targets the 18s rRNA of eukaryotic cells and shows a good fluorescence signal after hybridization to its target structure that is highly conserved among eukaryotes. In addition, the probe Non-EUB, a nonsense probe, was used as a negative control to screen for autofluorescence of fungal elements. All FISH probes were labelled with Cy5 and synthesized by EuroFins MWG/operon (Ebersberg, Germany). Characteristics of FISH probes are listed in Supplementary Table S1.

### 2.2. Evaluation of FISH Probes with Cultivated Fungi

Agents of mucormycosis (*Rhizopus oryzae*, *Lichtheimia corymbifera*, *Cunninghamella bertholletiae*, *Syncephalastrum racemosum*, *Mucor circinelloides*) and other fungi (*Aspergillus fumigatus*, *Candida glabrata*, *Candida parapsilosis*, *Lomentospora prolificans*) (Table 1) were cultivated in order to test for the probe’s sensitivity and specificity. All strains were grown for 6–24 h in RPMI medium at 37 °C for mold species (Aspergillus, *Mucorales* spp.) and at 30 °C for Candida until germlings and yeast cells were visible by microscopy. Cultivated fungi were washed with PBS, then fixed in 35% formalin overnight, washed three times, and stored in 50% ethanol. For hybridization, fungi were placed on Poly-L-lysine coated slides (Sigma-Aldrich, St. Louis, MO, USA) and treated with hybridization buffer consisting of 5 × SET (3.75 M NaCl, 25 mM ethylenediaminetetraacetic acid (EDTA), 0.5 M trisaminomethane (Tris), pH 7.8), 10% dextran, 0.2% bovine serum albumin (BSA), 0.1 mg/mL poly-adenosine, 20 μg/mL salmon testes deoxyribonucleic acid (DNA) (Sigma-Aldrich, St. Louis, MO, USA), 0.02% sodium dodecyl-sulfate (SDS) supplemented with 40 ng of FISH probes, and 20 ng 4’,6-diamidino-2-phenylindole (DAPI). Fungi were covered with hybridization buffer, coverslipped and incubated for 24 h at 50 °C in a humid chamber. After incubation, coverslips were removed by dipping in ice cold SET (1:5) buffer. Samples were washed with SET (1:100) buffer three times (10 min). Slides were dried and then covered with Vectashield anti-fade mounting media (Vector Laboratories, Burlingame, CA, USA), and a cover slip was applied for further analysis by fluorescence microscopy using a Zeiss Axio Imager Z1 Fluo-Microscope and imaging software ZEN (Zeiss, Jena, Germany) with constant exposure times.

The fluorescence signal of Mucorales-specific probes was compared with the positive signal obtained by the unspecific eukaryotic probe Euk516 and the negative control probe Non-EUB. A positive hybridization signal was defined as a higher signal as compared with nonsense probe Non-EUB, whereas fluorescence detected with Non-EUB and without probes was defined as autofluorescence. To confirm that the fluorescence was due to hybridization of the probes with its target structure rRNA, cultivated germlings and selected FFPET samples were treated with 5 μg/μL RNase dissolved in 10 mM Tris and 1 mM magnesium chloride prior to hybridization.

### 2.3. Mucormycosis Mouse Model

For evaluation of designed probes with tissue samples, a murine model for mucormycosis was utilized. Female Balb/c (16- to 18-week-old) mice were infected with *Rhizopus oryzae* (also known as *Rhizopus delemar*) (99–880) or *Mucor circinelloides* (1006PhL) via tail vein injection. To prepare inoculum, spores of the strains were suspended in sterile PBS and quantified using a hemocytometer. Two hundred microliters of the spore suspension (at 10^5^/mL for *R. oryzae* and 10^6^/mL for *M. circinelloides*) was used for injection. The animals were sacrificed 3–4 days post-infection, and the brains were collected and fixed in formalin before further procedure. FFPET brain sections were further processed for hybridization as described below for FFPET samples from patients with proven infection.

### 2.4. Clinical Samples of Patients with Invasive Fungal Infections 

FFPET specimens were obtained from 19 patients with invasive fungal infection, as demonstrated by fungal elements. Samples were chosen based on morphology of fungal elements in tissue to represent mucormycosis or other common deep mycoses, with or without detection of fungal DNA by broad-range PCR and sequencing (Table 2). 

DNA extraction from FFPE tissue was performed as described previously [5]. In short, three 5 μm slices of FFPET were placed in DNA-free tubes. Paraffin was removed with 99% octane for 30 s at room temperature. Next, DNA extraction was performed using the MasterPure™ yeast DNA Purification Kit (LGC Lucigen, Middleton, WI, USA) with two additional steps consisting of bead beating using Silicon-carbide sharp particles (Biospec Products Inc., Bartlesville, OK, USA) in a FastPrep-24™5G and a heating step (90 °C for 3 h) followed by incubation on ice (5 min). Extracted DNA was eluted in 75 µL Triton X 5%.

Fungal DNA was amplified by a broad-range qPCR assay targeting the 28S rRNA gene, as described previously [8]. Primers amplified a 330–350 bp sequence of a conserved region on the 28S rRNA using a SYBR^®^ green containing master mix. Amplification and melt curve analysis were performed using an Applied Biosystems™ 7500 Real-Time PCR System (Thermo Fisher Scientific Inc., Foster City, CA, USA). The PCR cycling conditions consisted of a 2 min uracil N-glycolase activation at 50 °C, followed by 10 min pre-melt at 95 °C and 45 cycles of 15 s melting at 95 °C, 30 s annealing at 55 °C, and 40 s extension at 72 °C. The melt curve was initiated with a 10 s step at 95 °C, then 5 s at 65 °C and a final 50 s at 95 °C. A positive broad-range PCR was defined as identical melting curves of amplified DNA for two replicates and amplification of a fungal DNA consistent with fungal elements in tissue, as identified by Sanger sequencing. All primers and probes were purchased from Eurofins Genomics (Ebersberg, Germany).

The hybridization procedure and fluorescence microscopy were performed as described above. FFPET specimens were scanned for fungal elements and for areas with positive hybridization signal. Performance of the hybridization procedure and fluorescence microscopy were performed by an investigator blinded to histopathology and PCR results.

The number of samples with specific FISH signal was compared to the number of samples amplifying Mucorales DNA among mucormycosis samples as detected by histopathology and were analyzed by two-sided Fisher’s exact test.

### 2.5. Assessing rRNA FISH Signal Intensity in Alternative Growth Conditions 

In order to identify potential reasons for diminished hybridization signals, *Rhizopus oryzae* germlings were exposed to a nutrient-deprived environment (PBS) and antifungal influence via amphotericin B (1 or 10 μg/mL). Germlings were formalin fixed after 0, 4, 8, 12, and 24 h of treatment and further evaluated via FISH for signal intensity. In order to identify fluorescence signal patterns in non-liquid media, *Lichtheimia corymbifera* was cultivated on RPMI containing Petri dishes at 37 °C for 48 h until colonies were formed. Selected colonies were cut out from the medium and embedded in liquid agar until agar solidified, then formalin fixed and paraffin embedded and cut for hybridization, as described for cultivated fungi.

## 3. Results

### 3.1. Generation of a Probe Combination Targeting Agents of Mucormycosis

Alignments of the 18S rRNA Gene sequences of agents of mucormycosis and other fungi suggested locations with a potential for selective targeting of most agents of mucormycosis. However, among sites of the rRNA gene previously documented to offer high signal intensity by FISH and conserved among all agents of mucormycosis, not a single probe was identified. Therefore, we aimed at a combination of probes targeting frequently identified agents of mucormycosis in Europe. We designed the probes “Mucorales” and “Lichtheimia” (Table S1) targeting the nucleotides 1101–1123 and 773–792 of the18S rRNA gene of *R. oryzae* (accession number AF113440). The Lichtheimia probe was designed because this fungus is a regular agent of mucormycosis in Europe. This fungus demonstrated a central mismatch with our Mucorales probe that resulted in weak fluorescence (data not shown). Of note, some sequences of *Apophysomyces* demonstrated one to two mismatches with the probes, suggesting that infections with these fungi may lead to suboptimal hybridization signals. Non-target fungi mostly demonstrated two to five mismatches in both probe sequences, suggesting a potential for specific hybridization signals.

**Figure 1:**
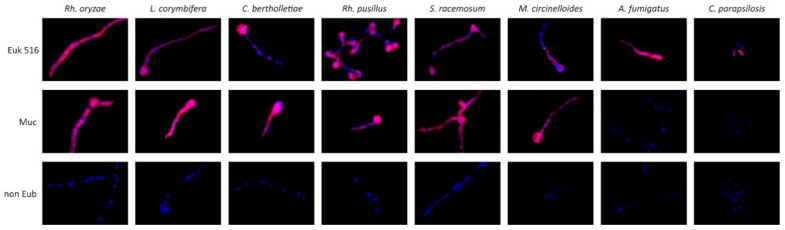
Evaluation of a combination of FISH probes selectively targeting the ribosomal RNA of agent germlings of frequent mucormycosis agents. Hybridization with the unspecific control probe targeting eukaryotic cells (Euk 516, upper) demonstrates a cytoplasmic signal of high intensity (upper row). Hybridization using a combination of probes targeting Mucorales demonstrates a cytoplasmatic signal comparable with the positive control but selective hybridization to Mucorales but not other selected agents of invasive fungal infections such as *A. fumigatus* and *C. parapsilosis* (central row). Hybridization with a nonsense probe (non Eub, lower row) demonstrating the absence of a fluorescence signal of fungal elements. All probes are coupled with Cy 5. Double stranded DNA stained with DAPI. Identical exposure times were used for each picture.

A combination of both probes in equimolar ratio showed a positive cytoplasmatic hybridization signal with the tested agents of mucormycosis but not with *A. fumigatus* (two mismatches) and *C. parapsilosis* (two mismatches) (Figure 1), *C. glabrata*, and *Lomentospora prolificans*. Most other fungi demonstrated two or more mismatches with both probes, respectively (Table S3). Fluorescence intensity with target fungi achieved with the probe combination resulted in a signal intensity comparable with EUK 516, the positive control probe with high signal intensity as demonstrated using Cy5-labeled probes. There was no autofluorescence detected by hybridization with the non Eub probe with all tested yeast cells and germlings of molds (Figure 1). 

### 3.2. Evaluation of the Mucorales Probe Combination with Mouse Tissue

**Figure 2:**
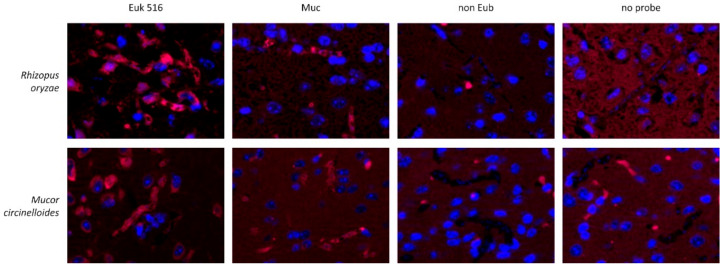
Evaluation of FISH probes targeting the ribosomal RNA of Mucorales with formalin-fixed, paraffin-embedded brain tissue from a mouse infection model. Mice were infected with either *Rh. oryzae* (upper) or *M. circinelloides* (lower). Hybridization with an unspecific probe targeting eukaryotic cells (Euk 516, first column) was used as a positive control with good cytoplasmatic signal intensity of Mucorales hyphae and host cells. Hybridization with the Mucorales probe combination (Muc, second column) demonstrated selective hybridization of hyphae from *Rhizopus oryzae* and *Mucor circinelloides* with signal intensity in the fungal cytoplasm comparable with the positive probe but not host tissue. Hybridization with a nonsense probe (non Eub, third column) or with hybridization buffer without FISH probe (last column) demonstrates the absence of any fluorescence signal of fungal elements in the cytoplasm and cell wall. All probes are coupled with Cy5. Double-stranded DNA stained with DAPI. Identical exposure times were used for each picture.

Hybridization of the probes targeting Mucorales with brain tissue from a mouse infection model demonstrated a fluorescence signal of the cytoplasm of *Rh. oryzae* and *M. circinelloides* with a fluorescence intensity comparable with the positive control. Hybridization with the nonsense probe or hybridization without any probe showed an absence of a signal in the fungal cytoplasm and of the fungal cell wall. Fungal nuclei demonstrated staining of double stranded DNA with DAPI (Figure 2). 

### 3.3. Evaluation of Mucorales Probe with Clinical Samples 

FFPET samples from patients treated for invasive fungal infections are summarized in Table 2. Histopathology suggested mucormycosis (n = 12), hyalohyphomycosis (n = 5), and candidiasis (n = 1). Fungal PCR and sequencing confirmed the fungal etiology, as identified by morphology of fungal elements in tissue in 13 samples (68%). Fungal etiology could not be confirmed due to negative PCR (n = 4) or mixed sequences preventing fungal identification (n = 2).

A positive hybridization signal identical to results seen with the tissue from the mouse models was detected with the positive control probe and the combination of probes targeting agents of mucormycosis in 7 of 12 samples (58%). However, in three of these samples, a cytoplasmatic fluorescence signal obtained with the nonsense probe or after hybridization without a probe suggested autofluorescence. Therefore, hybridization by FISH documented mucormycosis in 4 of 12 samples with hyphal elements was suggestive of mucormycosis, 8 of which were confirmed by amplification of DNA of Mucorales from the tissue sample (n.s.).

A positive hybridization signal with the positive control probe was detected in two of seven samples with hyalohyphomycosis or candidiasis. None of these samples showed a hybridization signal with the Mucorales probe combination.

**Figure 3:**
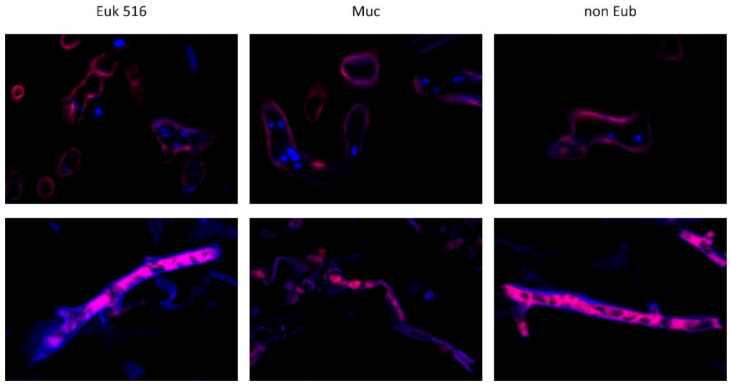
Unspecific fluorescence patterns in clinical samples with proven mucormycosis. Lack of cytoplasmatic hybridization signal (fluorescence in Cy5 channel) but fluorescence signal of fungal cell wall (Cy5 and Dapi channel) after hybridization with target probes and nonsense probe suggesting autofluorescence of the fungal cell wall (sample 11, upper row). Non-specific fluorescence in the DAPI and Cy5-channel of the cell wall and fluorescence signal localized in hyphal cytoplasm with all tested probes (sample 5, lower row). Fluorescence signal with nonsense probe non Eub and after RNAse treatment (pictures not shown) suggests that detected fluorescence within these samples is not due to hybridization of Cy5-labeled probes to the rRNA target.

Hybridization of the clinical tissue samples from mucormycosis demonstrated additional fluorescence microscopy patterns after hybridization, which were not seen in yeast cells, germlings of molds, or in tissue from the mouse model. First, a typical cytoplasmatic hybridization signal could not be identified in any of the hyphae in some tissue samples. Instead, fluorescence localized at the hyphal cell wall was detected. Fungal nuclei were detected in only some of the samples, whereas others appeared empty (Figure 3 upper row). Second, another fluorescence pattern found in FFPE samples was characterized by Cy5 fluorescence within the hyphal structures surrounded by a strong signal in the DAPI channel at the fungal cell wall (Figure 3, lower row). Fungal nuclear DNA indicated by granular DAPI-staining in the cytoplasm was not observed in these samples (Figure 3, lower row).

### 3.4. Assessing rRNA FISH Signal under Alternative Growth Conditions

**Figure 4:**
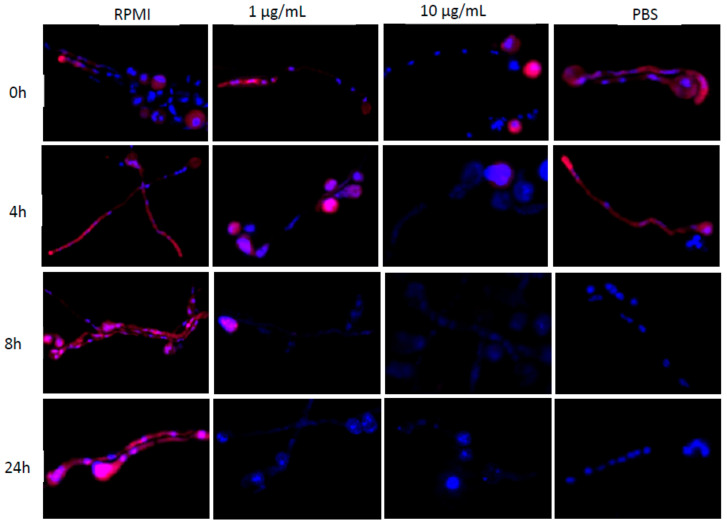
Nutrient deprivation and antifungal exposure reduce FISH signal of Mucorales hyphae. Hybridization of *R. oryzae* germlings with Mucorales-specific probes before and after exposure to amphotericin B (1 μg/mL, second column or 10 µg/mL, third column) or nutrient-limiting conditions (PBS) for up to 24 h. Positive hybridization signal of hyphae persists with continued incubation in RPMI (first column).

We hypothesized that the availability of nutrients in necrotic tissue or antifungal treatment may change fluorescence patterns as detected in tissue samples. We performed hybridization using the Mucorales-specific probe combination after 4 h, 8 h, 12 h, or 24 h incubation of *R. oryzae* germlings in PBS or RPMI containing 1 µg/mL or 10 µg/mL amphotericin B and compared fluorescence patterns with germlings with continued incubation in RPMI. Fungal cultures obtained after 4 h of media transfer already exhibited a loss in cytoplasmatic FISH signal when incubated in amphotericin B supplemented media that beam negative for the Mucorales-specific probe after 24 h (Figure 4, 3rd and 4th column). A strong hybridization signal remains in spores until 8 h of 1 μg/mL amphotericin B incubation and is completely ablated after 24 h. We could not detect a hybridization signal in all fungal structures from 4 h incubation onwards when treated with 10 μg/mL amphotericin B (Figure 4, 3rd column). Interestingly, we found irregularly shaped nuclei clusters in hyphae upon exposure to the minimal inhibitory concentration (MIC) of amphotericin B, which accumulated during the time course (Figure 4, 3rd column). Moreover, cultures treated with the 10-fold MIC of amphotericin B were marked by fewer and smaller spots of fungal DNA, which decreased over time (Figure 4, 4th column). PBS-incubated cultures presented a positive hybridization signal in both hyphal structures and spores until 8 h of nutrient deprivation (Figure 4, 4th column). The staining pattern of fungal nuclei present in hyphae of PBS cultures was similar to RPMI-incubated cultures. In contrast, cultures persistently incubated in RPMI showed a positive cytoplasmatic hybridization signal throughout all tested time points (Figure 4, 2nd column). 

To determine differences in hybridization signals across different parts of the mycelium, we embedded a 48-h-old colony of *A. corymbifera* in agar, which was then fixed in formalin and embedded in paraffin. Such agar-embedded cultures containing various hyphal stages were then further evaluated by FISH. Several hyphae displayed no cytoplasm-localized Cy5 fluorescence but rather strong DAPI-fluorescence restricted to the fungal cell wall (data not shown) comparable with the fluorescence pattern observed in infected human tissue samples. Furthermore, we detected FISH-positive hyphae containing DAPI-stained nuclei, which resembled hyphal structures found in Mucorales-infected mice (data not shown).

## 4. Discussion

Mucormycosis remains a serious threat to diverse patient groups [9,10]. Early specific antifungal management is needed for successful treatment outcomes [11]. Cultivation of Mucorales from clinical samples remains difficult, and identification of Mucorales by morphology of fungal elements in tissue has been shown to be error-prone. Consequently, experts identified the diagnosis of mucormycosis and the direct identification of fungal pathogens such as Mucorales in FFPE tissues as major diagnostic gaps [12].

We explored the application of fluorescence in situ hybridization (FISH) to identify mucormycosis in FFPE tissue samples. By using a combination of two Mucorales-specific FISH probes, we were able to selectively target the most frequent, phylogenetically distant agents of mucormycosis. Mucorales hyphae were identified in tissue samples from mouse models by a hybridization signal located to the fungal cytoplasm. However, the use of these probes as diagnostic tools for clinical samples might be limited by negative hybridization signals in some samples. Using in situ hybridization with non-fluorescently labeled probes has been previously used to identify fungal elements in patient samples. High specificities (94–100%) and more variable sensitivities had been documented for yeast infections (86%) and aspergillosis (96%). In this study, in situ hybridization for mucormycosis was less sensitive (79%) as compared with other fungal infections, although only samples from culture-positive biopsies were included in this study [4]. It is unclear why hybridization appears to be less successful in biopsies from mucormycosis. Using cultivated Mucorales hyphae, we showed that a fluorescence signal by probes targeting ribosomal RNA may be influenced by the availability of nutrients or the activity of antifungal agents. Further research may elucidate whether these patterns provide insights into the treatment response and by that improve management of patients with mucormycosis. Therapy of mucormycosis is often challenging, and expert opinion suggests that surgery should be considered in addition to antifungal therapy [2]. Autofluorescence documented in some clinical samples demonstrating hyphae but not in Mucorales germlings and in mouse tissue is a drawback to the use of FISH for diagnostic purposes on FFPE tissue. While autofluorescence can be detected by including nonsense probes, non-fluorescently labeled probes might be easier to use for diagnostic purposes. It is unclear which fungal molecules are responsible for autofluorescence in tissue biopsies from patients with mucormycosis. In addition, it is unknown whether autofluorescence occurs when using FISH with other clinical samples, such as respiratory secretions. 

The superiority of broad-range PCR for molecular detection of mucormycosis as compared with FISH has previously been suggested in a study using unspecific FISH probes. However, this study documented a PCR-negative and FISH-positive sample, implicating that the combination of both molecular tests improves sensitivity [5].

In conclusion, FISH using a probe combination targeting agents of mucormycosis may improve identification of these fungi in tissue. Different microscopic patterns observed in samples of proven mucormycosis by fluorescence microscopy after hybridization with FISH probes and DNA-binding dyes may represent adaptation of fungi in the host, the availability of nutrients, and response to antimicrobials. Further studies may help to understand these patterns in order to determine whether they can be used to infer treatment response of patients with mucormycosis. 

## Figures and Tables

**Table 1:** Fungal isolates used for experimental evaluation of FISH probes targeting common agents of mucormycosis. All strains were identified by macro- and micromorphology and sequencing of the complete internal transcribed spacer (ITS) region.

**Agents of Mucormycosis**	**Strain Number**
*Lichtheimia corymbifera*	11–0171
*Cunninghamella bertholletiae*	09–0536
*Rhizopus oryzae*	10–077
*Mucor circinelloides*	13–1227
*Syncephalastrum racemosum*	13–1228
**Non-Mucorales Species**	
*Aspergillus fumigatus*	11–0169
*Candida glabrata*	ATCC 64677
*Candida parapsilosis*	DSM 11225
*Scedosporium prolificans*	04–0286

Strains without letters are from the strain collection of the Robert Koch Institute.

**Table 2:** Formalin-fixed, paraffin-embedded tissue samples from patients with proven fungal infection as diagnosed by histopathology and broad-range fungal qPCR.

Sample No.	Euk	Non Eub	Mucorales Probes	No Probe	Fungal PCR and Sequencing (Histopathology)
1	+	-	+	-	*Rhizopus* sp.(M)
2	+	-	+	nt	*Rhizomucor* sp. (M)
3	+	-	+	nt	*Rhizopus* sp. (M)
4	+	-	+	-	*Rhizopus* sp. (M)
5	+	+	+	+	*Rhizopus* sp. (M)
6	+	+	+	+	mixed sequence (M)
7	+	+	+	+	*Rhizopus* sp. (M)
8	-	-	-	-	*Lichtheimia* sp. (M)
9	-	-	-	-	negative (M)
10	-	-	-	-	mixed sequence (M)
11	-	-	-	-	*Rhizopus* sp. (M)
12	-	-	-	-	negative (M)
13	-	-	-	-	*A. fumigatus* (H)
14	+	-	-	-	*C. glabrata* (C)
15	-	-	-	-	negative (H)
16	-	-	-	-	*A. fumigatus* (A)
17	-	-	-	-	*S. boydii* (H)
18	+	-	-	nt	*A. flavus* (H)
19	-	-	-	-	negative (H)

Euk: positive control probe targeting eukaryotic 18s rRNA, Non Eub: nonsense probe, Mucorales probes: combination of Mucorales and Lichthaemia probe targeting agents of mucormycosis, all probes were labeled with Cy5, no probe refers to hybridization with hybridization buffer without probe. + indicates specimens with fluorescence of fungal elements detected in the Cy5 channel; - are samples with no fluorescence signal in Cy5 channel, samples not tested under this condition are labelled as nt, M: Mucormycosis; H: Hyalohyphomycosis; C: Candidiasis.

## Supplementary Materials

The following are available online at https://www.mdpi.com/article/10.3390/jof8030289/s1, Table S1: Characteristics of FISH probes, Table S2: Mismatches of the probes with fungi of the Zygomycetes, Table S3: Mismatches of probes with 18s rRNA of non-target fungi.
